# Overview of Respiratory Sensor Solutions to Support Patient Diagnosis and Monitoring

**DOI:** 10.3390/s25041078

**Published:** 2025-02-11

**Authors:** Ilona Karpiel, Maciej Mysiński, Kamil Olesz, Marek Czerw

**Affiliations:** 1 Lukasiewicz Research Network-Krakow Institute of Technology, Zakopiańska 73, 30-418 Kraków, Poland; maciej.mysinski@kit.lukasiewicz.gov.pl (M.M.); kamil.olesz@kit.lukasiewicz.gov.pl (K.O.); marek.czerw@kit.lukasiewicz.gov.pl (M.C.); 2 Institute of Biomedical Engineering, Faculty of Science and Technology, University of Silesia in Katowice, 39 Będzińska, 41-200 Sosnowiec, Poland; 3 Department of Biosensors and Processing of Biomedical Signals, Faculty of Biomedical Engineering, Silesian University of Technology, 44-100 Gliwice, Poland

**Keywords:** respiratory sensor, respiratory monitoring devices, wearable respiratory sensors, telemedicine in respiratory care

## Abstract

Between 2018 and 2024, the global market has experienced significant advancements in sensor technologies for monitoring patients’ health conditions, which have demonstrated a pivotal role in diagnostics, treatment monitoring, and healthcare optimization. Progress in microelectronics, device miniaturization, and wireless communication technologies has facilitated the development of sophisticated sensors, including wearable devices such as smartwatches and fitness trackers, enabling the real-time monitoring of key health parameters. These devices are widely employed across clinical settings, nursing care, and daily life to collect critical data on vital signs, including heart rate, blood pressure, oxygen saturation, and respiratory rate. A systematic review of the developments within this period highlights the transformative potential of AI and IoT-based technologies in healthcare personalization, particularly in disease symptom prediction and public health management. Furthermore, innovative techniques such as respiratory inductive plethysmography (RIP) and millimeter-wave radar systems (mmTAA) have emerged as precise, non-contact solutions for respiratory monitoring, with applications spanning diagnostics, therapeutic interventions, and enhanced safety in daily life.

## 1. Introduction

A noticeable trend is the growing popularity of wearable devices, such as smartwatches and fitness bands, which can simply connect to a smartphone—a device most people have with them at all times. These devices allow for the measurement of basic health parameters, increasing people’s awareness of health. There are also wearable devices, which are medical devices that, through connections to dedicated applications, allow a doctor to monitor health in real time and take faster action on required critical situations. The widespread use of miniaturized and wearable sensors plays a key role in medicine, enabling the remote monitoring of patients by doctors. With the help of such medical devices, doctors can remotely observe health conditions, make precise diagnostic decisions, and adjust therapies according to the current state of health. They do not have to be guided by past results, only, instead, by the current state.

One of the key issues is the diagnosis of the respiratory system, and the available devices and sensors allow for different observations of respiratory parameters depending on the needs. Using the available devices [1,2,3,4,5], we can monitor parameters such as respiratory rate (RR), tidal volume (TV), oxygen saturation (SpO2), chest movements, and periods of apnea and hypopnea. These devices also allow for the monitoring of sleep, respiratory rhythm, and changes in body position. Recent years have shown an increase in the importance of respiratory diagnostics for general health assessments, but also for sudden exacerbations of disease. Selecting appropriate diagnostic techniques can help to highlight respiratory problems in specific cases. Various sensors and techniques will be used, for example, to check the status of an asthma exacerbation, to monitor a patient during sleep, or to observe patient parameters in the intensive care unit.

Moreover, the development of artificial intelligence (AI) has enabled more sophisticated analysis of the data collected by health sensors. AI can help to identify patterns and detect abnormalities in health parameters, which can lead to faster diagnosis and intervention.

With the increase in health data collection, there are also concerns about data security and patient privacy. Companies and organizations must take precautions to protect this information from unauthorized access. As this technology grows, regulations and standards are also being introduced to ensure the safety and effectiveness of health sensors. Organizations, such as the Food and Drug Administration (FDA) in the United States, are implementing regulations for the registration and approval of such devices. The health sensor market is growing rapidly, and more and more companies are investing in this field. Competition and innovation are contributing to the rapid introduction of new products and lower costs.

## 2. Materials and Methods

**Figure 1:**
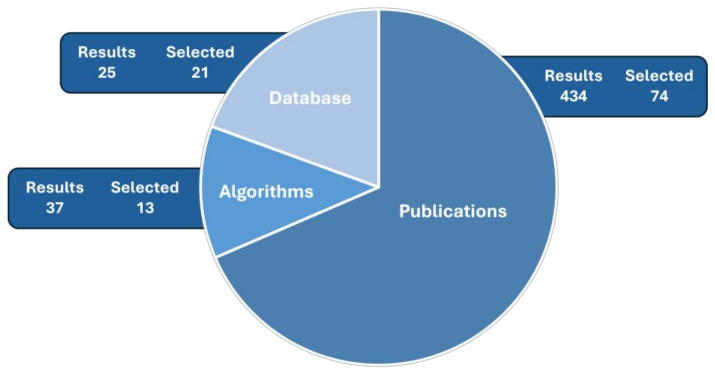
Tally of search results and selected records for publications, databases, and algorithms.

This study reviewed the available solutions that emerged between 2018 and 2024, focusing on monitoring or diagnosing patients with upper respiratory tract conditions. The review was conducted without the use of AI tools for generating comparisons. The search for articles was conducted in the IEEE Xplore database between 9 October and 31 October 2024, using terms related to machine learning, artificial intelligence, and respiratory monitoring techniques. Additionally, the GitHub platform was searched for repositories of databases related to respiratory monitoring, which provided additional resources supporting the development of new AI-based solutions. As a result, a full-text review of 111 articles was conducted, from which 77 publications were selected that best met the review criteria. Our team focused on the IEEE Xplore database, considering it to be the most suitable for engineering and technological research, offering more relevant and up-to-date resources compared to databases like PubMed, Scopus, or Web of Science, which focus on other fields. Studies in the review were selected at all stages by three analysts working independently, as shown in Figure 1.

## 3. Open Database

**Figure 2:**
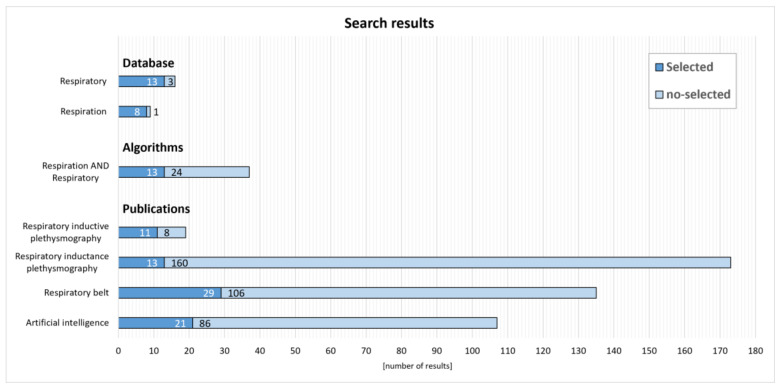
Characteristics of the results obtained from the PhysioNet and IEEE database in response to selected keywords from 1 January 2018–27 November 2024.

In today’s world, medicine and information technology are closely collaborating, leading to increased accessibility and the exchange of medical data. International organizations are developing standards and protocols to facilitate the exchange of medical data between different information systems. Interoperability is crucial for the global exchange of medical information. There is a noticeable trend indicating a growing demand for data, particularly physiological signals, respiratory signals, respiratory parameters, and others. Figure 2 presents the number of responses to a query related to signals in the publicly available PhysioNet database. The database was searched and the most relevant keywords were “respiration” and “respiratory,” which together form a dataset of 21 results (with one dataset appearing twice).

Databases containing signals are essential in the context of modern medicine and information technology because they are key to modern diagnostics and patient monitoring. They are crucial for the development and validation of AI algorithms, and the PhysioNet database provides the actual data needed to train machine learning algorithms. The keyword “Respiration” appears relatively infrequently and the reviewed databases, although widely used, do not respond 100% to market demand and the needs of researchers who are in the process of designing new devices.

The characteristics (I) of the bases in response to the keyword “respiration” are as follows:

- Simultaneous physiological measurements with five devices at different cognitive and physical loads: Measurement included electrocardiography (ECG), photoplethysmography, accelerometry, oxygen saturation respiration, heart rate (HR), heart rate variability (HRV), and RR intervals ranging from 1 Hz to 8000 Hz [6].
- MIMIC-IV waveform database: The MIMIC-IV waveform database is a collection of physiological signals and measurements from patients in intensive care units, including electrocardiograms, photoplethysmograms, respiratory parameters, blood pressure measured invasively and non-invasively, and other measurements. These measurements and signals are obtained directly from the bedside monitor and provide a detailed picture of the physiology of critically ill patients [7].
- NInFEA: Non-Invasive Multimodal Fetal ECG-Doppler Dataset for Antenatal Cardiology Research: The first publicly available dataset of simultaneous non-invasive electrophysiological recordings, fetal pulsed-wave Doppler (PWD), and maternal respiratory signals [8].
- Electrocardiogram, skin conductance and respiration from spider-fearful individuals watching spider video clips: This project includes an electrocardiogram, skin conductance, and respiration as raw data (unfiltered, unprocessed) recorded from consenting individuals afraid of spiders using a BITalino portable biosignal measurement device (PLUX—Wireless Biosignals SA, Lisbon, Portugal) with a sampling rate set to 100 Hz per channel at a resolution of 10 bits [9].
- MIMIC-III waveform database: The MIMIC-III waveform database contains 67,830 sets of records for approximately 30,000 intensive care unit patients. The majority of record sets include a waveform record containing digital signals (usually including ECG, ABP, respiration, and PPG) and a ‘numerical’ record containing a time series of periodic measurements, each representing a quasi-continuous record of vital signs [10].
- MIMIC-III waveform database matched subset: Matched subset of the ‘MIMIC-III waveform database’. Matches records with the older clinical database ‘MIMIC-III clinical database’. Contains 22,317 waveform records and 22,247 numerical records for 10,282 different ICU patients [11].
- Cerebral Vasoregulation in Elderly with Stroke: Contains multimodal data from a large study investigating the effects of ischemic stroke on cerebral vasoregulation. The cross sectional study compared 60 subjects who suffered strokes to 60 control subjects, collecting the following data for each patient across multiple days: transcranial Doppler of cerebral arteries, 24 h blood pressure numerics, high resolution waveforms (ECG, blood pressure, CO_2_, and respiration) during various movement tasks, 24 h ECG, EMG (electromyography), accelerometer recordings, and gait pressure recordings during a walking test [12].
- BIDMC PPG and Respiration Dataset: Dataset contains signals and numerics extracted from the larger MIMIC II matched waveform database, along with manual breath annotations made from two annotators using the impedance respiratory signal [13].

The second characterization (II) of the bases in response to the keyword “respiratory” is as follows:

- Pressure, flow, and dynamic thoraco-abdominal circumferences data for adults breathing under CPAP therapy: This database of respiratory pressure, flow, and dynamic chest and abdominal circumferences was collected from 30 healthy adults at the University of Canterbury. Measurements were made using a bidirectional Venturi taper and a tape measure with rotary encoders, which recorded chest expansion and contraction at different pressures and breathing conditions. The data were intended to validate respiratory monitoring systems for use in primary care and home settings [14].
- Cerebral perfusion and cognitive decline in type 2 diabetes: This collection includes studies on vasoregulation and blood flow involving 70 patients with type 2 diabetes and 70 healthy control patients aged 50–85 years [15].
- Cerebromicrovascular Disease in Elderly with Diabetes: This database from a prospective study evaluates the effects of type 2 diabetes on cerebral vasoregulation, perfusion, and functional outcomes in 69 participants aged 55–75 with diabetes and in a control group. Data include measurements of blood flow, pressure, heart rate, respiratory parameters, gait, balance, and MRI images, including CASL, FLAIR, and DTI [16].
- CPAP Pressure and Flow Data from a Local Trial of 30 Adults at the University of Canterbury: The study includes pressure and flow measurements during breathing with CPAP (Continuous Positive Airway Pressure) from 30 patients [17].
- Upper body thermal images and associated clinical data from a pilot cohort study of COVID-19: The database contains upper-body thermal recordings from 252 patients in the COVID-19 study who performed breath-holds in four positions. The data include PCR results, demographics, vital signs, activities, medications, respiratory symptoms, and respiratory rate. The aim of the study was to analyze temperature patterns for developing algorithms to analyze thermal recordings [18].
- MIMIC-III and eICU-CRD: Feature Representation by FIDDLE Preprocessing: This collection of data is derived from the MIMIC-III and eICU databases, and includes the features and labels for five prediction tasks related to three adverse outcomes: in-hospital mortality (48 h), acute respiratory failure (4 h, 12 h), and shock (4 h, 12 h) [19].
- Cerebral Vasoregulation in Diabetes: A database of studies evaluating the effects of type 2 diabetes on cerebral vasoregulation and body function. It contains data from 37 diabetics and 49 control subjects aged 55–75, with continuous measurements of cerebral blood flow (TCD, MRI), heart rate, blood pressure, respiratory parameters, balance, gait, and laboratory results [20].
- OpenOximetry Repository: The OpenOximetry database contains clinical and laboratory data on pulse oximetry, including high-frequency waveforms, oxygen saturation readings, and other physiological parameters. It enables research on the impact of physiological variables on pulse oximeter performance [21].
- A Temporal Dataset for Respiratory Support in Critically Ill Patients: The dataset includes 90-day hourly respiratory parameters, such as ventilation data, breathing support interventions, and other parameters from 50,920 ICU patients. The data are sourced from MIMIC v2.2 and also contain laboratory results and therapy details [22].
- Respiratory and heart rate monitoring dataset from aeration study: The dataset includes respiratory pressure and flow, electrical impedance tomography (EIT), ECG, and heart rate belt (HRB) data collected from 20 healthy individuals. Participants breathed through a full-face mask with a pressure gauge and flowmeter [23].
- Simulated Obstructive Disease Respiratory Pressure and Flow: The dataset includes respiratory pressure and flow parameters collected from 20 healthy adults simulating COPD effects, such as gas trapping during exhalation. Tests were conducted using a Venturi splitter with a device attached to the oral exhalation outlet [24].
- Respiratory dataset from PEEP study with expiratory occlusion: The dataset includes respiratory pressure and flow, dynamic chest and abdominal circumference data, and aeration from EIT, which were collected from 80 adults. Participants breathed with CPAP support, and the study was conducted with approval from the University of Canterbury HREC [25].

In addition to the databases appearing in the PhysioNet database, several databases were found that also answer our query and can provide the necessary signals:

- Cough Database contains samples of coughing and other breathing-related sounds. It is a useful source of data for research on respiratory problems [26].
- Polysomnographic databases include the SHHS (Sleep Heart Health Study) or NSRR (National Sleep Research Resource), which contain data related to breathing during sleep [27,28].

Projects are also underway to collect a large amount of data:

- Chronic Obstructive Pulmonary Disease Gene “COPDGene” is a multi-center research project to understand and investigate the causes and mechanisms of chronic obstructive pulmonary disease (COPD), a serious respiratory condition [29].

## 4. Algorithms

A sizable portion of the algorithms, programs, and databases that are made available on the Internet and used by the whole world are on the GitHub platform. Table 1 shows the databases used in the development of new solutions based on AI. The review of repositories was carried out from 9 October 2024 to 31 October 2024. The keyword “Respiration” on the GitHub platform appears 566 times, and “Respiratory” yields 4300 results. Narrowing the search to “Topics”, the word “Respiration” obtains 23 results, and “Respiratory” 14. Other items were not included in the review.

Unfortunately, the content is not verified, despite the fact that it is a popular way of posting data. There is a lack of order, verification, and review. A sizable part of the content of repositories is empty or contains only part of the code, without descriptions and reliable information. It is worth mentioning that the selected keywords are also related to other topics besides human respiration analysis algorithms, i.e., soil respiration, database intermediary programs, visualization programs (without analysis), libraries for handling integrated circuits, converter schemes, and more. This ambiguity complicates the search for relevant materials and diminishes the usability of these repositories for researchers and developers. To address these challenges, there is a growing need for a structured approach to organizing and verifying data within such repositories. Implementing a standardized method for documenting and reviewing contributions could significantly enhance the quality of shared resources. One promising solution is the concept of the ’Shared Open Network’ (SON), which emphasizes collaboration, verification, and transparency among users. By fostering a community-driven environment, SON can facilitate the effective exchange of high-quality information, making it easier for researchers to find credible data and tools tailored to their specific needs. In this way, we can strive toward a more reliable and efficient landscape for data sharing and analysis in various fields, including human respiration studies. In Table 1, we present solutions that appear on platforms such as GitHub, blogs, and other open source sources, but none of them are medical devices that could be tested under real-world conditions that could be implemented shortly. Some solutions have been in existence for more than 10 years, and they are still being developed and modified. Still, they are not for the evaluation of physiological parameters in real conditions, including the hospital or home.

## 5. Results

We conducted a systematic search in one electronic database: IEEE explore. We searched for articles published between January 2018 and October 2024. The search was conducted between 9 October and 31 October, 2024. The reference lists of the included articles were reviewed to check for possible articles that could be included. Regarding search terms, the search strategies applied differed depending on the nature of the databases chosen for the search. For example, PubMed allows for the application of limiters such as “humans” and “English” language articles. The terms identified were (“Artificial Intelligence” OR “Respiratory Belt” OR “Respiratory Inductance Plethysmography“ OR “Respiratory Inductive Plethysmography”, OR “ Respiratory Sensor”), which are presented in Figure 2.

We reviewed the full text of 434 articles. The selection process aimed to ensure the inclusion of diverse and high-quality resources, focusing on technical and engineering-oriented solutions in respiratory monitoring. The review may vary depending on the database selected and the keywords chosen by the specialists. Our team chose to review the IEEE Xplore database, which is managed by the Institute of Electrical and Electronics Engineers (IEEE), which focuses mainly on the literature in engineering, computer science, telecommunications, and related technologies. This database was prioritized due to its extensive repository of peer-reviewed articles that align with the technical aspects of our research goals, offering insights into cutting-edge innovations in IoT, AI, and sensor-based respiratory monitoring. Our choice is based on the conviction that IEEE Xplore is a better choice for technical, engineering, and IT research and projects, offering more relevant, up-to-date and industry-specific resources compared to PubMed, which focuses on biomedical sciences. We skipped searching databases such as Scopus and Web of Science, focusing only on one specific one. The table is divided into 4 sections and includes the 74 publications our team selected, taking into account the abbreviated description of the method. To ensure clarity in data categorization, publications were grouped based on their focus areas, including disease detection, AI integration, and real-time monitoring applications.

Six papers were repeated in response to queries, and six did not fit into our review and were omitted from the review paper description. The review focused on solutions that were dedicated to various conditions, applied or prepared for diagnosis and later therapy, and the real-time monitoring of patients at home and in facilities. Particular emphasis was placed on evaluating the practical application and readiness level of these technologies in clinical and non-clinical environments. There are so many similar solutions, as it turns out, that keywords do not necessarily allow for a precise narrowing of the topic. The abundance of overlapping solutions highlights the challenge of distinguishing innovative methodologies from incremental advancements, necessitating rigorous criteria for inclusion and detailed analysis. Table 2 shows the results, the areas of which fall into six main groups, i.e., vital signs monitoring [48,49], disease detection and diagnosis [50,51], application in diagnostic breathing sounds [52,53], innovative technologies in disease detection [54,55], use of AI in the COVID-19 pandemic [56,57], and, in general, the challenges and future of AI in health monitoring.

**Table 2:** Summary of scientific publications on respiratory sensor solutions for patient diagnosis and monitoring.

Authors (Year)	Methods	Description
	Parameters	
Keywords: Respiratory Belt [Index Terms, Autor Keywords, IEEE Terms]		
Coronel et al., 2021 [58]	3D Camera, SpO2 desaturation.	Using the respiratory signal from a 3D camera, a new algorithm was developed that detects reduced respiratory motion and SpO2 desaturation to evaluate respiratory events
	Respiratory motion.	
He et al., 2020 [59]	“Kinect” sensor, IR-UWB Radar.	A system for monitoring respiratory rate in a room using three impulse radio ultra-wideband (IR-UWB) radars and a Kinect camera. The IR-UWB radar system covers the entire room, allowing for the respiratory monitoring of individuals regardless of their orientation relative to the radar. The Kinect camera tracks 3D joint coordinates, enabling the precise localization of individuals. The results demonstrated high effectiveness in tracking both single and multiple subjects and in estimating respiratory rate.
	Respiratory motion.	
Kusche et al., 2022 [60]	32-channel differential EMG.	A novel non-invasive respiratory monitoring device using a dry electrode belt for capturing diaphragm EMG signals from the thorax.
	Respiration through thorax movement.	
Vanbuis et al., 2022 [61]	Cardio-respiratory polysomnography, tracheal sound sensors.	The three-step sleep scoring model for Type III sleep studies was trained and tested using 300 and 100 independent recordings from patients suspected of having sleep breathing disorders, with the steps as follows: classification using a multi-layer perceptron, correction of sleep transition rules according to AASM guidelines, and sequence correction using a Viterbi hidden Markov model.
	Sound, respiration, heart rate, oxidation.	
Alam et al., 2019 [62]	Electronic circuit with a push button (Arduino) integrated into a chest belt.	A non-invasive, wearable solution to track and analyze respiratory rate in real time. The circuit detects chest movements associated with breathing, and the push button registers these movements to calculate and display the respiratory rate via a Bluetooth application.
	Respiratory rate.	
Qiu et al., 2021 [63]	Two-electrode bioimpedance (BioZ) sensor.	It can be used for the accurate monitoring of breathing rate in both static and dynamic conditions, including the calculation of the respiratory rate. The sensor measures the change in chest impedance resulting from breathing. Additionally, an integrated medical-grade infrared temperature sensor allows for body temperature measurement. The recorded data are transmitted via a Bluetooth module to a computer for processing and visualization.
	Respiratory rate.	
Sharma et al., 2019 [64]	Radio frequency (RF) sensor (near-field coherent sensing) placed near the xiphoid process.	The sensor uses an RF signal at a frequency of 1.8 GHz, which is modulated by the movement of internal organs and then received and demodulated by the receiving antenna. This sensor enables the monitoring of breathing rate and lung volume by detecting peaks in the respiratory cycle.
	Respiratory rate, lung volume.	
Laufer et al., 2020 [65]	Thoracic belt to measure changes in the circumference of the chest or abdomen.	This belt allows for the accurate monitoring of changes in body circumference, enabling the determination of respiratory parameters such as breathing rate. The measurement results were compared with a motion capture system, which served as a reference.
	Respiratory rate, tidal volume.	
Nedoma et al., 2018 [66]	Optical interferometric sensor based on fiber-optic and connected to an optical interrogator.	Measures respiratory rate and pulse rate and allows for the improvement of MR images of the head, compensating the respiratory motion. The sensor is encased in a thin layer of polyurethane, which provides immunity to electromagnetic interference (EMI) and allows its use in environments with magnetic resonance imaging (MRI).
	Respiratory rate, heart rate.	
Shakhih et al., 2018 [67]	Infrared thermal imaging camera (ITI).	Uses an infrared thermal imaging camera (ITI) for thermal imaging to assess the timing of inspiration (TI) and expiration (TE) during prolonged expiration breathing.
	Respiratory rate.	
Nassi et al., 2022 [68]	Polysomnography, artificial neural network.	Uses a neural network (WaveNet) to analyze 9656 polysomnographic recordings from Massachusetts General Hospital (MGH). It detects obstructive apnea, central apnea, hypopnea, and respiratory effort-related arousals.
	Respiratory rate, apnea.	
Tataraidze et al., 2019 [69]	Wi-Fi signal.	Analyzes channel status information (CSI) from the Wi-Fi connection between the smartphone and the access point for breath detection.
	Respiratory rate.	
Wang et al., 2021 [70]	Breathing sensor in the form of a piezoresistive matrix.	It is used to monitor force distribution and dynamic resistance waveforms for respiratory rate analysis. The matrix (composed of graphite and silver paste) is placed on the mattress to record changes in the pressure exerted on it during breathing.
	Respiratory rate.	
Erdyarahman et al., 2022 [71]	A non-contact radar system using frequency-modulated continuous wave (FMCW) technology.	The radar uses frequency-modulated continuous wave (FMCW) technology to monitor small movements in near real time. Electromagnetic waves are emitted by a transmitting antenna (Tx), reflected off a target (e.g., chest), and received by a receiving antenna (Rx).
	Respiratory rate.	
Cruz et al., 2021 [72]	Pulse oximeter, artificial neural network.	The selected five pulse rate features, namely, time of peaks, peaks, time of valleys, valleys, and time since the last peak, provide a proper estimation of RR. The neural network helps in the classification of the labels of valleys such as inspiration, expiration, and neutral.
	Respiratory rate through heart rate.	
Bevilacqua et al., 2022 [73]	Piezoelectric belt, data acquisition is via a DAQ board.	The algorithm takes advantage of an adaptive bandwidth filter to identify the breathing condition and to quantitatively determine the respiratory rate. The algorithm detects moments of dyspnea by identifying outliers. Początek formularzaDół formularza
	Respiratory rate.	
Guede-Fernandez et al., 2019 [74]	Respiratory inductance plethysmography (RIP) belt.	Drowsiness detection is based on the real-time analysis of respiratory rate variability (RRV) using an RIP belt and signal quality assessment to reduce the number of false alarms associated with body movements. The method includes signal quality assessment to reduce false alarms caused by body movements, rather than drowsiness.
	Respiratory rate.	
Sanae et al., 2022 [75]	A sensor in the smart seatbelt buckle (SSB) to detect driver drowsiness based on breath duration.	The accuracy of the calculated breath time obtained using the smart seatbelt buckle (SSB) was assessed in comparison to reference respiratory sensors. Subsequently, based on this breath time, an attempt was made to estimate the drowsiness level during driving simulation, achieving a high correlation and indicating the possibility of assessing a drowsiness level of 4 or higher.
	Respiratory rate.	
Padasdao et al., 2018 [76]	Non-contact detection of chest circumference change.	The sensor uses a modified direct current (dc) motor as an electromagnetic (EM) generator, mounted on the chest, to record changes in chest circumference and tidal volume (TV), while simultaneously generating energy to power the sensor.
	Respiratory rate, tidal volume.	
Nedoma et al., 2022 [77]	Breath sensor (FOBM, pneumatic respiratory belt) in magnetic resonance (MR).	The article described the testing of fiber-optic Bragg grating embedded in an oxygen breathing mask (FOBM) in a real MR environment using 3 T imaging, as well as the comparison of results with other methods, such as the navigator technique and pneumatic respiratory belt. The FOBM approach provides reliable and accurate results, with an overall image quality score of 3.3 ± 0.4 for FOBM, compared to 3.7 ± 0.5 for the Siemens reference system and 3.8 ± 0.2 for respiratory belts.
	Breath peaks.	
Härmä et al., 2023 [78]	Deep learning, audio, RIP sensor.	Predicts the next moment of inhalation based on the speaker’s speech using deep learning techniques.
	Inhalation moment.	
Zhang et al., 2021 [79]	Radio frequency (RF) sensor integrated in furniture, near-field coherent sensing (NCS) technique.	A breath sensor integrated into a bed or chair that detects breathing by modifying the structure of a radio frequency (RF) coaxial cable with a designed notch and utilizes the near-field coherent sensing (NCS) technique. The sensor records respiratory waveforms and determines the breathing rate, and it can also assess the heart rate in the same configuration with appropriate filtering. The sensor design is engineered to tolerate significant positional variation, allowing it to adapt to different user positions.
	Respiratory waveform, respiratory rate, heart rate.	
Mukhopadhyay et al., 2018 [80]	A wearable IoT-based sensor. A pressure-sensitive textile fabric attached to a belt.	Real-time respiratory signal monitoring. The converted-to-electric-signal chest/stomach movement is transmitted to a central base station, where it is displayed in real time and uploaded to an online repository for future analysis.
	Respiratory waveform.	
Addeh et al., 2023 [81]	Elastic band around abdomen, fMRI.	There is no guarantee that a given respiratory event evident in the abdominal respiratory belt transducer timeseries, such as a deep breath or pause in breathing, will be detectable in both respiratory volume per time (RVT) and respiratory variation (RV). In addition, RVT and RV do not show similar behavior during some respiratory events, especially when the subject breathes deeply at a low rate, while they use similar respiratory response functions.
	Respiratory rate, breathing pattern.	
Piuzzi et al., 2019 [82]	A 1-channel bioimpedance and ECG using a one or two pair electrode configuration.	The measurement performance was compared in two-electrode and four-electrode configurations, assessing the sensitivity of heart activity detection and comparing the results with a respiratory belt and an ECG device.
	Respiration rate and waveform through bioimpedance, ECG.	
Erdogan et al., 2019 [83]	A 24 GHz microwave Doppler sensor.	A network system was developed to gather data from distributed nodes, simultaneously monitoring multiple patients and tracking parameters such as temperature, pressure, and humidity. The system also enables real-time monitoring of cough and apnea and provides warning signals to caregivers in emergency situations.
	Respiratory rate.	
Altekreeti et al., 2021 [84]	Chest belt with force–pressure sensor.	A neonatal apnea detection system utilizing a sensor embedded in a soft smart e-textile chest belt that is integrated with a smartphone app.
	Respiratory rate.	
Zhao et al., 2020 [85]	Doppler radar sensor, depth camera.	Non-contact respiration detection using the fusion of a Doppler radar sensor and depth camera. The proposed fusion scheme, using a Bayesian fusion algorithm, can provide more accurate respiratory rate estimation compared to using a single sensor.
	Respiratory rate.	
Nabavi et al., 2020 [86]	Direct oral cavity pressure sensor.	Tracks respiratory patterns and detects respiratory events regardless of airway, i.e., nasal and oral. Minimal susceptibility to motion artifacts. Direct measurement of oral pressure with 99% accuracy compared to a reference measurement.
	Respiratory rate, breathing pattern.	
Nallanthighal et al., 2021 [87]	Respiratory belt, microphone.	Analysis of breathing belt data and phoneme-matched audio data.
	Respiratory waveform, speech signal.	
Kjar et al., 2021 [88]	Plethysmography belt.	Analysis of respiratory and chest movement patterns using plethysmography belt signals to distinguish between obstructive and central sleep apnea.
	Polysomnography (PSG).	
Li et al., 2023 [89]	Four-dimensional imaging radar using a variational mode separation (VMS) algorithm to separate respiratory and cardiac signals.	The proposed VMS algorithm separates the weaker cardiac motion pattern from the stronger respiratory motion pattern, reducing the influence of respiratory harmonics on the heart signal.
	Respiratory rate, heart rate.	
Torres et al., 2019 [90]	RIP sensor, mobile app.	The system uses a sensor on a belt, a Raspberry Pi to process the data, and an HTTP server to upload the results to the mobile app. The device has been proven to be effective for people who are uncomfortable using a mouthpiece, and provides a convenient tool for initial lung diagnosis.
	Respiratory rate, tidal volume.	
Slastnikov et al., 2021 [91]	Review and analysis of methods for detecting the human respiratory wave: MRI with a respiratory belt; the acoustic method; polysomnography using a thermistor and nasal pressure transducer; and implementations of respiratory wave detection based on video signals from RGB, RGB-D, and thermal cameras.	The features of video-based respiratory wave detection in newborns are discussed, and a selected method for the video-based detection of this wave is presented.
	Respiratory waveform.	
Islam et al., [92]	Microwave Doppler radar.	Microwave Doppler radar was used for respiration monitoring, and the feasibility of separating the respiratory signals from multiple subjects was explored using ICA with the JADE algorithm. The method successfully separated signals from two subjects 1 m apart at a distance of 2.89 m from the radar.
	Respiratory waveform.	
Valentina et al., 2018 [93]	Respiratory belt, 14-channel electroencephalogram (EEG), 6-channel electrogastrogram (EGG), 1-channel electrooculogram (EOG), 1-channel electrocardiogram.	The study measured brain, eye, cardiac, respiratory, and gastric activity using EEG, EOG, ECG, a respiratory belt, and EGG. Subjects, after fasting, watched film clips with different emotional content, followed by undergoing a water load test. Results showed increased gastric activity in response to emotional stimuli, suggesting central nervous system (CNS) to enteric nervous system (ENS) inhibition, aligning with animal model findings.
	Breathing pattern, EGG, EEG, EOG, ECG.	
Sacco et al., 2020 [94]	Frequency-Modulated Continuous Wave (FMCW) radar	The 5.8 GHz FMCW radar, specifically designed for measuring respiratory rate and heartbeat, was tested in four orientations of the subject relative to the antenna. Results were compared with photoplethysmograph and respiratory belt measurements, demonstrating high accuracy in all scenarios.
	Respiratory rate, heart rate	
W. Kang et al., 2024 [95]	FMCW radar (Frequency-Modulated Continuous Wave) with a metasurface antenna and passive metasurface tags.	Monitors the breath of the driver and passengers in a car. The use of metasurface tags improves the signal-to-noise ratio and enables the precise differentiation of the breaths of different individuals in the presence of interference inside the vehicle.
	Respiratory rate.	
G. Deshpande et al., 2023 [96]	The microphone built into a smartphone.	Extracts breath patterns from speech signals recorded using a smartphone’s microphone. The study showed that different categories of breath can be identified based on speech signals with a classification accuracy of 79%.
	Breathing pattern through speech.	
Keyword: Respiratory Inductance Plethysmography [ALL]		
Elfaramawy et al., 2019 [97]	The Inertial Measurement Unit (IMU) and microphone.	The wireless system uses wearable sensors with a low-power 9-axis IMU and MEMS microphone to monitor breathing and coughing rates in real time. Data processing algorithms calculate respiratory frequency and coughing events. Tests show high accuracy compared to respiratory inductance plethysmography.
	Respiratory rate, breathing pattern, cough rate.	
Bricout et al., 2019 [98]	Adaptive Accelerometry Derived Respiration (ADR).	The ADR method uses two 3-axis accelerometers to monitor breathing by analyzing chest and abdominal motions. Compared to measuring airflow through the nose, the method achieves 74% agreement, suggesting a high accuracy in assessing respiratory rate and volume.
	Respiratory rate, tidal volume.	
Bin Nesar et al., 2022 [99]	LiDAR (Light Detection and Ranging) system, multi-pixel thermal sensor.	The system consists of a rotating LiDAR scanner and a multi-pixel thermal sensor. It is used to determine both respiratory rate and tidal volume, and enables posture assessment and the separate evaluation of nasal and mouth breathing.
	Respiratory rate, tidal volume.	
Sadr et al., 2019 [100]	Electrocardiogram (ECG), respiratory induction plethysmography (RIP).	ECG and RIP signals were analyzed in different combinations. The QRS area and Principal Component Analysis (P CA) methods were used to estimate ECG-derived respiratory (EDR) signals and Cardiopulmonary Coupling (CPC) spectra. Classification of sleep apnea was performed using Linear Discriminant Analysis (LDA).
	Respiratory rate, tidal volume.	
Hurtado et al., 2020 [101]	RIP belt, temperature sensor, machine learning, Random Forest regression.	Temperature and breathing signals were used to process temperature-related features to train machine learning models to predict TV (tidal volume) and MV (minute ventilation). The best results were obtained with Random Forest regression, achieving minimal error for TV and MV.
	Tidal volume, minute ventilation.	
Huysmans et al., 2020 [102]	Tachogram, RIP sensor, convolutional neural network (CNN).	Tachogram data and respiratory inductive plethysmography (RIP) signals were used to classify sleep using a 1D convolutional neural network (CNN) to classify 30 s epochs and to detect and classify sleep apnea.
	Respiratory rate, heart rate.	
Mannee et al., 2020 [103]	RIP sensor in a smart shirt.	A smart t-shirt with an inductive sensor measures changes in the transverse circumference of the chest and abdomen. It was used to monitor lung hyperinflation (LH). The effects of temperature and girth on the sensor data were tested. The results showed a linear relationship between temperature and RIP signal, and confirmed a linear relationship between girth and sensor signal.
	Respiratory rate, lung hyperinflation.	
Hill et al., 2021 [104]	LiDAR system and thermal sensor in a breathing mask for non-contact breath monitoring.	The system measures changes in respiratory mask temperature and chest and abdominal movements using a thermal sensor and LiDAR system. The results were compared with data from RIP, capnometry, spirometry, and pulse oximetry. A high correlation was obtained between LiDAR measurements and tidal volume values and capnometer data.
	Respiratory rate, tidal volume.	
Guo et al., 2019 [105]	Flexible Tactile Sensor Array on a mattress.	A system of tactile sensors on a bed was used to distinguish between chest and abdominal movements by measuring pressure changes on the mattress. Tests were conducted, comparing the results with respiratory induction plethysmography (RIP). The sensor showed a high accuracy in measuring respiratory rate, and also noted differences in the phase of pressure changes depending on gender and lying position.
	Respiratory rate.	
Song et al., 2023 [106]	A bed-sized tactile sensor sheet.	A bed-sized tactile sensor sheet was used to monitor and identify chest and abdominal position and movements during sleep. Optimal measurement areas were studied based on the distribution of body pressure on the mat. A suitable mathematical model and discriminative feature procedure were proposed, which improved the accuracy of the measurements and the usefulness of the method in the early detection of respiratory diseases.
	Respiratory rate, respiratory pressure distribution.	
Rathore et al., 2022 [107]	RIP sensor, residual fluctuation analysis (DFA).	The utility of respiratory rate (BR) for determining the ventilatory threshold (VT1) was studied in comparison with classical methods based on gas analysis and heart rate (HR). Residual fluctuation analysis (DFA) and respiratory induction plethysmography (RIP) were used to validate the model.
	Respiratory rate, ventilatory threshold.	
Nabavi et al., 2023 [108]	A sensor based on surface acoustic waves (SAW) near the nose or mouth.	The SAW sensor for continuous breath monitoring, based on humidity measurements, was characterized by high sensitivity and precision in determining the respiratory rate and breath patterns. Its effectiveness has been confirmed through a comparison with a traditional breath monitoring belt.
	Respiratory rate, breathing pattern.	
Akamatsu et al., 2023 [109]	Face image analysis based on video recordings.	The CalibrationPhys method was applied, which enabled the measurement of heart rate and respiratory rate through the analysis of facial images in video. This method uses synchronized recordings from multiple cameras and employs contrastive learning to predict pulse and breath waves.
	Respiratory rate and heart rate through facial video.	
Keyword: Respiratory Inductive Plethysmography [ALL]		
Senyurek et al., 2019 [110]	RIP sensor, AI algorithms.	The inductive sensor monitors changes in chest volume associated with smoking. The study compared the effectiveness of deep learning algorithms, such as convolutional neural networks (CNNs) and Long Short-Term Memory (LSTM), with traditional methods, such as support vector machines (SVMs), Markov models, and decision trees, in detecting smoke inhalation.
	Tidal volume.	
Schulz et al., 2018 [111]	RIP sensor, 64-channel EEG, 3-channel ECG.	The study monitored heart rate (BBI), respiratory rate, and power EEG (PEEG) from a 64-channel EEG in 21 healthy subjects to analyze interactions occurring in the Central Cardiovascular and Respiratory Network (CCRN).
	Respiratory rate, heart rate, EEG, ECG.	
Azimi et al., 2018 [112]	Dual RIP sensor.	The method uses the sum of signals from two RIP sensors as an alternative to the airflow signal in detecting apnea and shallow breathing events.
	Respiratory rate, apnea.	
Mateu-Mateus et al., 2020 [113]	Optical camera with computer algorithms, RIP sensor.	The method uses a camera to track chest movements from a lateral perspective. The algorithm analyzes the consecutive frames of the video in gray tones, which reduces the computational cost and allows for real-time analysis. In addition, a signal quality index is determined.
	Breathing pattern.	
Belsare et al., 2020 [114]	RIP sensor.	Personal Automatic Cigarette Tracker v2 (PACT-2) is a system that uses an RIP sensor to analyze respiratory signals. It measures parameters such as the duration of inhalation and exhalation, as well as respiratory volume, providing new indicators of the depth and timing of smoke inhalation.
	Respiratory rate, tidal volume.	
Lyakhova et al., 2018 [115]	Bioimpedance and plethysmography using inductive and piezoelectric sensors.	The wireless device records bioimpedance, inductive plethysmography, and piezoelectric signals, synchronizing the measurements. It analyzes the data in MATLAB, processing the signals and calculating the main relevant parameters.
	Respiratory rate, respiratory wave.	
Huang et al., 2021 [116]	RIP sensor.	The induction sensor measures chest wall and abdominal movements during the spontaneous breathing test (SBT). The instantaneous phase difference (IPD) method assesses the asynchrony of these movements, supporting extubation.
	Respiratory rate, spontaneous breathing trial.	
Huang et al., 2018 [117]	RIP using the instantaneous phase difference (IPD) method.	The RIP sensor measures tidal volume, and the instantaneous phase difference (IPD) method assesses the synchronization between chest wall movements (TMV) and abdominal wall movements (AWM) in patients with COPD.
	Tidal volume.	
Zhu et al., 2019 [118]	Infrared camera and video analysis	The use of an infrared camera and analysis of the recordings tracks the movement of selected feature points on the body of a sleeping patient and extracts the respiratory rate using independent component analysis.
	Respiratory rate.	
SolĂ-Soler et al., 2023 [119]	Pneumotachograph, RIP belt.	Pneumotachograph and inductive plethysmography belts for breath measurement.Comparison of the breathing pattern characteristics obtained from sensors, pneumotachograph, and inductive plethysmography belts was used to assess breathing variability in healthy volunteers.
	Breathing pattern.	
Zhang et al., 2024 [120]	Millimeter-wave radar (mmWave Radar), AI, RIP for reference.	The mmTAA system, based on millimeter-wave (mmWave) technology, is used for the non-invasive measurement of TAA (Target Angle and Azimuth) and for detecting and monitoring breath without physical contact. It utilizes an mmWave radar with multiple antennas and an advanced neural network, TAANet, which allows for the accurate determination of the centroid position of RC-AB, enabling precise measurements.
	Breathing pattern.	
Keyword: Artificial Intelligence AND (respiratory signal OR respiratory rate) [Index Terms, Autor Keywords, IEEE Terms]		
Karvounis et al., 2021 [48]	Prototype wrist sensor.	The wrist sensor monitors real-time vital signs such as heart rate, respiratory rate, oxygen saturation, temperature, and changes in systolic blood pressure. The collected data are sent to a cloud-based environment, where it can be processed using machine learning techniques or Information Mining Algorithms. Automatic alerts are sent to medical personnel, enabling quick intervention.
	Respiratory rate, temperature, SpO2, PPG, ECG, blood pressure, motion.	
Chen et al., 2021 [49]	Barometric sensor and signal processing algorithm.	A barometric sensor placed on a desk in a person’s environment monitors pressure changes caused by breathing and coughing. A signal processing algorithm is used with a sparsity-based filter. It effectively detects coughing and assesses the respiratory rate, achieving 97.33% accuracy in detecting coughing and 98.98% specificity in assessing breath.
	Respiratory rate, respiratory waveform, breathing pattern, cough detection.	
Jiang et al., 2022 [50]	ECG, 9-axis MEMS IMU sensor, PPG.	The impact of the COVID-19 virus on overall health was discussed, and existing vital sign monitoring systems and their limitations were analyzed. Potential options for estimating lung function using sensor fusion and artificial intelligence (AI) techniques were also explored. The prototype device demonstrated performance comparable to or better than similar commercial devices.
	Respiratory rate through ECG, lung volume through IMU sensor, heart rate, cough detection, temperature.	
Ghimire et al., 2020 [121]	**An overview of the applications of artificial intelligence, machine learning, and deep learning in solving COVID-19 pandemic problems.**	An overview of the use of artificial intelligence, machine learning, and deep learning in the context of the COVID-19 pandemic. Applications included diagnostics, mortality forecasting, vaccine and drug development, sentiment analysis of comments on COVID-19, and disinformation detection. The review included the most successful models in the field.
	**Not specified.**	
Fakotakis et al., 2023 [122]	Acoustic sensor, machine learning techniques.	A monitoring system equipped with an acoustic sensor and sound detection was discussed for recognizing drug activation and assessing medication adherence in patients with asthma. The article presented the use of machine learning techniques in the Respiratory and Drug Actuation suite (RDA) for sound processing, feature extraction, and classification.
	Sound detection for recognizing drug activation.	
Elias et al., 2021 [123]	Acoustic collar, thermoelectric sensor, AI.	A neck device equipped with acoustic and thermoelectric sensors, linked to an artificial intelligence algorithm, was used for the non-invasive assessment of pathophysiological status and the dynamic observation of lesions resulting from COVID-19 infection. The device effectively distinguished between patients with mild symptoms and those with acute symptoms of respiratory failure.
	Sound detection of vocal system.	
Chawla i Walia, 2022 [52]	AI algorithms to analyze lung images, respiratory sounds, and medical textual data.	Artificial Intelligence based Techniques in Respiratory Healthcare Services: A Review
	Not specified.	
Husain et al., 2022 [124]	Review of AI technology for COVID-19 disease detection using cough, breath, and speech recordings.	The review included 24 studies and eight applications using artificial intelligence algorithms to detect COVID-19 from audio recordings of coughs, breathing, and speech. AI-based methods could prove effective in screening and diagnosing respiratory diseases, which could save time and play an important role in the fight against COVID-19 and future pandemics.
	Not specified.	
Yahyaoui i Yumusak, 2021 [125]	AI algorithms for detecting respiratory diseases	Deep And Machine Learning Towards Pneumonia And Asthma Detection
	Not specified.	
Ward et al., 2021 [126]	Long-wave infrared (LWIR) face detection, deep convolutional network (DNN), cough sounds.	The COVID-19 non-contact symptom detection device uses LWIR face detection to monitor facial temperature and an acoustic sensor and light convolutional network to determine whether a person is coughing. The system was tested on two datasets: one with infrared images of the face and the other with audio recordings of coughing, confirming its effectiveness in detecting symptoms.
	Not specified.	
Dong i Yao, 2021 [56]	Internet of Things (IoT) technology to monitor physiological parameters, including breathing.	The paper described the application of IoT technology for respiratory monitoring in the context of the COVID-19 pandemic, discussing various methods such as inertial sensors, thermal cameras, optical cameras, microphones, radar, and WiFi. An IoT platform for monitoring COVID-19 was presented, including symptoms, quarantine, contacts, social distance, epidemic forecasting, and virus mutation.
	Not specified.	
Misra et al., 2023 [57]	KEdge analytical platform (fuzzy interference system).	KEdge is an advanced analytics platform that uses IoT sensor data to assess patient health by analyzing physiological parameters such as respiratory rate, ECG signals, pulse rate (PR), blood oxygen saturation (SpO2), and blood pressure. The system uses a two-stage analytical process and a fuzzy inference system (FIS) to assess the severity of cardiac and respiratory conditions and the health condition index (CI).
	Respiratory rate, ECG, heart rate, SpO2, blood pressure.	
Campana et al., 2022 [127]	L3-Net deep embedding model for automatic analysis of audible cough and breath samples to identify patients with COVID-19	L3-Net, based on Transfer Learning, enables COVID-19 detection from smartphone breath and cough recordings. The model, trained on 2 million audio recordings, effectively extracts hidden audio features, which are analyzed by SVM classifiers and logistic regression. It is suitable for deployment on mobile devices.
	Not specified.	
Abdullah i Bilal Er, 2022 [51]	Hybrid convolutional neural network (CNN)–Long Short-Term Memory (LSTM) model for lung signal classification	The model combines CNN and LSTM structures to extract features and classify lung sounds as normal or abnormal. It uses Cosine Similarity-based Multilevel Discrete Wavelet Transform Decomposition (CS-MDWTD) and the Butterworth filter for noise reduction, and then classifies sounds using CNN-LSTM networks. The model was tested on the Respiratory Sound Database (ICBHI 2017), achieving a classification accuracy of over 90%.
	Not specified.	
Rani et al., 2021 [53]	Machine learning classifiers: support vector machine (SVM), k-Nearest Neighbors (KNN), Naïve Bayes Classifier, and artificial neural networks (ANN); acoustic features of lung sounds.	Automatic lung sound classification model using artificial intelligence (SVM, KNN, Naïve Bayes, ANN) to diagnose lung diseases. The method identifies various differentiating features of lung sounds, such as the sound of wheezing, which can be useful in the diagnosis of asthma. The model achieved an average accuracy of 95.6%.
	Lung sounds.	
Grooby et al., 2023 [54]	An artificial intelligence-based model using non-negative matrix factorization (NMF) and non-negative matrix cofactorization (NMCF).	Noisy Neonatal Chest Sound Separation for high-quality heart and lung sound separation of neonatal chest sounds based on artificial intelligence NMF and NMCF methods.
	Chest sounds.	
Kuang et al., 2023 [55]	Remote photoplethysmography (rPPG) technology, facial video.	rPPG technology is used to measure physiological indicators based on facial video, including HR and respiratory rate (RR). Remote photoplethysmography signal are enhanced based on generative adversarial networks.
	Respiratory rate, heart rate.	
Romero Gomez i Orjuela-Canon, 2021 [128]	ANN, machine learning techniques, sound signal.	The use of artificial intelligence to analyze respiratory sounds, such as crackles and wheezes, which are associated with respiratory diseases. AI algorithms are used to classify these sounds based on the Respiratory Sound Database from the ICBHI 2017 challenge.
	Not specified.	
Türkçetin et al., 2023 [129]	ANN, sound signal.	The use of artificial intelligence to analyze sound signals, such as cough, breath, and the sound /a/, for diagnosing respiratory diseases (asthma, COPD, pneumonia).
	Breath detection, cough detection.	
Pouramirarsalani et al., 2024 [130]	The microphone built into a mobile phone.	The use of a microphone and mobile phone to record acoustic signals related to breathing during sleep, which are then analyzed for sleep apnea diagnosis. The classification model based on artificial intelligence and optimization using the PSO algorithm enabled effective diagnosis, achieving a classification accuracy of 87.5%.
	Sleep apnea.	
Saeed et al., 2023 [131]	Radar technology and Wi-Fi (radar systems based on SDR).	Utilization of radio technologies, including radar and SDR (software-defined radio), for detecting breath patterns. The radar system tracks subtle chest movements associated with breathing, while SDR systems analyze Wi-Fi signals, enabling the accurate classification of breath patterns using deep learning algorithms, achieving a high classification accuracy
	Breathing pattern.	

Each of these categories represents a pivotal area of respiratory monitoring, with implications for both individual patient care and public health. The use of AI and IoT solutions in the COVID-19 pandemic, for instance, demonstrates their scalability and adaptability in large-scale health crises. As AI and IoT technologies develop, the potential for using these tools to personalize healthcare is growing. Examples shown in Table 2, such as the use of AI to analyze audio and image signals, point to the potential to create advanced predictive algorithms that can forecast the onset of disease symptoms or predict the risk of disease exacerbation. These predictive capabilities are particularly relevant for chronic respiratory diseases, where early intervention can significantly impact patient outcomes. For instance, algorithms capable of detecting subtle changes in lung sounds could serve as early warning systems for exacerbations in COPD patients. Relying on multi-modal data—including information on heart rhythm, breathing, lung sounds, or signals from motion sensors—could enable a more comprehensive assessment of a patient’s health. This could lead to personalized therapy tailored to individual patients, which is particularly important for chronic diseases, such as chronic obstructive pulmonary disease (COPD) or diabetes. The integration of multi-modal data, as evidenced in the reviewed studies, not only enhances diagnostic accuracy but also opens pathways for developing holistic health monitoring platforms that address comorbidities. Another important aspect is the possibility of using combined AI and IoT systems to manage public health. Solutions such as IoT-based platforms for monitoring and controlling the COVID-19 pandemic show that similar systems can be adapted to monitor other infectious diseases or the broader population health. By leveraging real-time data aggregation and analytics, these systems can facilitate proactive measures, such as the early identification of outbreaks and resource optimization during healthcare crises. Automated collection and analysis of data on symptoms, test results, and human contact information can support a rapid response to potential outbreaks, which is crucial in the context of future pandemics. Such capabilities exemplify the shift towards data-driven public health strategies, which rely on AI and IoT integration for scalability and precision.

Respiratory induction plethysmography (RIP) is a modern and non-invasive method of monitoring respiratory patterns that is increasingly used in the analysis of smoking behavior. A key aspect of a respiratory sensor in diagnostics and monitoring is its ability to accurately and continuously record breathing patterns, allowing for the detection of abnormalities and early identification of potential health risks, such as airway obstruction or apnea. Thanks to advanced data analysis algorithms, the information collected by the sensors—which includes parameters such as breathing rhythm, tidal volume, and the synchronization of chest and abdominal movements—can be processed in real time. This makes it possible to create predictive algorithms that support both the personalization of therapy and monitoring in home and clinical settings. In addition, integration with AI and IoT technologies allows for the analysis of multi-modal data, which improves the accuracy of diagnoses and opens up new possibilities in the management of chronic diseases such as COPD and asthma. This technique uses inductive sensors to measure changes in the volume of the thorax and abdomen, which allows for detailed monitoring of respiratory parameters such as tidal volume (TV), respiratory rate, and the duration of inhalation and exhalation phases. In the context of smoking, RIP enables precise detection of respiratory patterns associated with smoke inhalation, which can be crucial in assessing its impact on the respiratory system.

In recent years, the effectiveness of RIP has been significantly improved by the use of advanced artificial intelligence algorithms [132,133,134,135] such as convolutional neural networks (CNN) and Long Short-Term Memory (LSTM). Specifically, CNNs excel in identifying spatial patterns in respiratory data, while LSTMs are particularly effective in modeling temporal dependencies in respiratory cycles, making them ideal for dynamic applications like detecting irregular smoke inhalation patterns, demonstrating improved sensitivity and specificity in complex scenarios, such as distinguishing smoking behavior from other respiratory events. Studies have shown that these deep learning algorithms outperform traditional methods such as support vector machines (SVM), Markov models, or decision trees in detecting smoke inhalation patterns, suggesting their potential in the precise monitoring of smokers’ behavior. Moreover, the method has a wide range of applications in clinical diagnostics, including monitoring the synchrony of chest and abdominal movements in patients with chronic obstructive pulmonary disease (COPD), assessing respiratory asynchrony during the spontaneous breathing test (SBT), and detecting apneas and shallow breathing.

Despite its many advantages, RIP is not without its limitations. Inductive sensors are susceptible to interference from non-respiratory body movements, which can affect the quality and accuracy of data. In addition, this technology requires precise calibration and proper positioning of sensors on the patient’s body, which can be challenging in a home setting. Advanced algorithms that incorporate motion artifact correction and dynamic re-calibration techniques have been proposed to address these issues, enhancing the reliability in non-clinical environments. However, the development of more advanced filtering and correction algorithms, as well as integration with other measurement methods such as heart rate (HR) monitoring and electroencephalography (EEG), can significantly improve the quality and versatility of RIP applications [136]. The use of RIP in combination with bioimpedance and piezoelectric signal analysis also allows for a more comprehensive assessment of the patient’s health status, which may be particularly important in the context of integrated cardiorespiratory monitoring.

As the technology evolves, the introduction of more compact, wireless systems, such as the Personal Automatic Cigarette Tracker v2 (PACT-2), and the integration of RIP with optical and infrared methods of chest movement analysis, may revolutionize the way respiratory health is monitored, both in clinical and home settings [137,138]. Furthermore, hybrid systems that combine RIP with advanced radar technologies offer promising avenues for creating low-cost, contactless solutions capable of delivering comparable accuracy to traditional methods.

The latest developments in radar technology raise significant and impactful issues for modern applications. In 2024, Kang et al. [95] described the advanced use of Frequency Modulation Continuous Wave (FMCW) radar technology combined with a metasurface antenna and passive metasurface tags, designed to monitor the breathing of the driver and passengers in a car. In the context of contemporary challenges, such as road safety and increasing vehicle automation, this solution holds great potential for accident prevention. For instance, it can detect symptoms of driver fatigue or health issues like sleep apnea, enabling timely interventions. Moreover, the ability to precisely track passengers’ breathing in real time offers opportunities for the development of intelligent climate control systems or personalized cabin settings, significantly enhancing travel comfort. This innovation also paves the way for medical diagnostics while driving and the seamless integration with autonomous vehicle systems, fostering a safer and more intuitive driving environment. Similarly, Zhang et al. [120] introduced the mmTAA system in 2024, which utilizes millimeter-wave (mmWave) radar with multiple antennas and an advanced neural network, TAANet, for the non-invasive measurement of Target Angle and Azimuth (TAA) and monitoring of respiratory activity. This system excels at accurately determining the centroid position of respiratory chest and abdominal movements (RC-AB), achieving precision comparable to traditional methods, such as Optoelectronic Plethysmography (OEP). The mmTAA system demonstrates immense potential for revolutionizing respiratory health monitoring in daily life by providing a non-contact, reliable solution. Its practical implementation in wearable and stationary devices could bridge the gap between clinical-grade accuracy and everyday usability. Its ability to continuously track respiratory parameters in a discreet and user-friendly manner could significantly improve the early diagnosis and management of respiratory conditions, such as respiratory insufficiency or sleep disorders. Together, these innovations showcase the transformative power of radar technology in enhancing safety, comfort, and health monitoring in modern vehicles and daily life. By integrating advanced signal processing, metasurface designs, and machine learning, they address critical challenges in mobility and healthcare, pointing towards a future where technology seamlessly enhances both safety and well-being.

### 5.1. Development of Respiratory Monitoring Technologies for Infants and Children

This review also helped to locate a niche of sorts when it comes to the use of similar devices that will support respiratory monitoring, but in MRI, fMRI or rsfMRI studies [81]. Monitoring respiration in infants and young children poses significant challenges due to the irregularity of their breathing patterns and the various physiological conditions that can affect monitoring accuracy. In infants, particularly in the first months of life, there are notable fluctuations in breathing rates, a result of the immaturity of the respiratory and autonomic systems. Research has shown that infants can exhibit breathing rates ranging from 30 to 60 breaths per minute, with distinct periods of rapid breathing interspersed with slower breaths. For instance, studies such as those conducted by Njeru et al. [139] and Arzi et al. [140] document the variability in infant breathing and the impact of sleep on respiratory patterns. The inclusion of real-time monitoring tools that adapt dynamically to these fluctuations is critical for ensuring reliable assessments during sleep studies or developmental evaluations. This variability, often exacerbated during sleep, can be a critical indicator of respiratory health and development in infants.

### 5.2. From Non-Specific Methods to Dedicated Solutions

The transition from non-specific methods to dedicated solutions in respiratory monitoring illustrates the versatility of engineering devices originally designed for unrelated tasks. Many technologies, such as buttons used in input devices or radios [62,64], have been tested in experimental setups to assess their feasibility for respiratory analysis, despite their lack of direct implementation in clinical environments. Similarly, motion-sensing technologies like Kinect [59], initially developed for gaming and motion detection, have shown potential when repurposed for monitoring respiratory patterns. These methods, while not inherently intended for respiratory analysis, have been adapted with some success due to their ability to capture relevant physiological data.

Further, technologies dedicated to a single function, such as sensors used exclusively for sleep monitoring, are being progressively refined and modified for real-time respiratory monitoring applications. An example includes their evolution from tracking sleep-related events to providing continuous feedback during day-to-day activities. Additionally, respiratory detection is increasingly used to support other modalities, such as magnetic resonance imaging (MRI), where respiratory signals [66] are employed to minimize motion artifacts by synchronizing image acquisition with periods of breath-hold or reduced motion.

Even non-respiratory tools, like pulse monitors, have been leveraged to extract respiratory parameters such as breathing rate, demonstrating how multipurpose data can enhance clinical insights. Meanwhile, proximity-based devices like LiDAR [99], which require the subject to remain within a specific field of detection, highlight the range of contactless solutions emerging for respiratory monitoring. These devices complement traditional approaches, and are often validated against gold-standard methods like plethysmography, where new techniques are benchmarked against established respiratory measurement tools [118]. This trend showcases the potential of flexible, multi-functional engineering methods in advancing respiratory diagnostics beyond their original intent.

### 5.3. The Need for Advanced Monitoring in a Medical Context

Monitoring respiration in children is crucial not only for assessing their development, but also for the early detection of potential health risks, such as Sudden Infant Death Syndrome (SIDS) and asthma. Studies indicate that infants are particularly vulnerable to SIDS in the early months of life, and early respiratory monitoring can help to identify the risk and potential preventive interventions [141,142]. Furthermore, children with asthma and allergies may experience breathing difficulties, necessitating the precise monitoring of their respiratory patterns to tailor therapy and manage symptoms effectively [143,144]. Integrating modern respiratory monitoring technologies, such as remote sensing and real-time analysis, can provide valuable data and enhance care for children with specific health needs.

## 6. Conclusions

Devices in the form of wearable respiratory belts can be utilized in various contexts, including sleep monitoring, patient assessment, and even sports training. Their capability to track respiratory parameters offers valuable insights into respiratory health and can aid in the early identification of potential issues. As technology evolves, ongoing comparisons of available solutions are essential to ensure that theoretical advancements keep pace with practical developments. This continuous evaluation will facilitate the refinement of current solutions and the emergence of novel approaches, thus driving innovation in respiratory monitoring.

The integration of AI and IoT in respiratory monitoring systems holds significant promise for personalized healthcare and precision diagnostics. Key areas for future research include optimizing analytical algorithms for the more accurate detection of respiratory changes and the early identification of health risks. The combination of different technologies—such as breath monitoring, sound analysis, and imaging—may enable the development of advanced diagnostic and predictive systems.

One of the challenges that remains is improving the accuracy of monitoring in home settings, particularly in minimizing disturbances caused by body movements. The miniaturization of wearable devices, along with the integration of multimodal data (e.g., heart rate, breath patterns, sound signals), is expected to enhance personalized therapies, particularly for chronic conditions such as COPD. Furthermore, the integration of these systems with public health monitoring platforms can contribute to rapid responses in the face of health crises, such as pandemics.

While these advancements are promising, further studies are needed to address issues related to data privacy and security. Additionally, regulatory frameworks and user education will be crucial to ensuring the safe and responsible implementation of respiratory monitoring technologies in clinical practice.

## Figures and Tables

**Table 1:** Algorithms, databases, and software sources hosted on the Github platform.

Name	Description	Programming Language/Environment	Author
Rrest [30,31]	The algorithm estimates the respiratory rate from ECG or PPG. It analyzes frequency (Fourier) and checks the time between successive ECG heartbeats or PPG beat episodes. It then modulates the signal in three ways—changing the baseline, amplitude, or frequency.	Matlab	Peter Charlson
Neonatal-Respiration-Monitoring-Algorithm [32,33]	The algorithm estimates the respiratory rate based on a video (individual, consecutive frames). A convolutional neural network searches for abdominal movements in the area of interest (ROI) and estimates the respiratory rate (RPM) based on them. The algorithm is designed for videos of infants lying in care units (works with good focus and lighting). It works only on hdf5 files.	Python	Adam Nagy
ALT [34,35]	Embedded on a Raspberry Pi, the system includes a camera and a light source (sub-black) with a depth-of-field detector. The collected image frames are processed by the AI—ALT “superimposes” a texture (dots) on the person, mapping the positions of the various body parts, then checks the differences in the positions of the superimposed dots for each successive frame. In this way, it checks, for example, chest movements both through breaths and small areas with a visible heartbeat. It is mainly used to monitor vital signs during sleep.	Python	Alexander Misharin
Real-time visual respiration rate estimation with dynamic scene adaptation [36,37]	The algorithm focuses on the real-time estimation of respiration rate based on video analysis. It uses image processing and machine learning techniques to track chest and abdominal movements, even in changing scene conditions, such as variations in lighting or a person’s position. The algorithm is designed to work in different environments, making it useful for remote health monitoring.	Python/Matlab	Mayank Mishra
respirationCA [38]	The algorithm analyzes how phases of the respiratory cycle and moments in the cardiac cycle affect tactile perception. It synchronizes (phase-locking) respiratory signals with expected stimuli to identify moments of peak heart rate and associated higher alertness. The analysis reveals that tactile detection is lowest when the pulse wave reaches the finger and highest during diastole. This suggests that these changes are not merely physiological artifacts but result from cognitive processes that model the body’s internal state and may adjust respiration to fit the task at hand.	Matlab/R	Martin Grund
ecg_respiration_sleep_staging [39]	Deep neural networks are developed to classify sleep stages using the ECG and respiratory signals from 8682 polysomnographs. Five types of networks are trained to analyze ECG R-peaks and respiratory effort.	Python + PTH models	Harvard Medical School
RF_respiration_monitoring [40]	The algorithm uses radio frequency signals to monitor respiration. It processes reflected RF waves to detect breathing movements and extract respiratory patterns. The system is designed for real-time monitoring, offering continuous updates on the breathing rate and patterns.	Matlab + simulink	isuparnopal
UWB_Radar_Respiration_Monitoring [41,42]	The algorithm uses ultra-wideband (UWB) radar to monitor respiration rates. It processes radar signals to detect and analyze chest movements caused by breathing. The algorithm extracts respiratory patterns from the radar data and calculates the respiratory rate. This non-invasive method enables the real-time monitoring of respiration without physical contact.	Python, Matlab	Minsun Kim
Infant-Respiration-Estimation [43]	The algorithm estimates infant respiration rates from video data. It uses computer vision techniques to analyze subtle chest movements associated with breathing. The algorithm processes these movements to accurately determine the respiratory rate in real time. This non-invasive method is designed to monitor infant breathing without the need for physical sensors.	Python	Sarah Ostadabbas (Northeastern University)
rPPG_Toolbox [44]	The algorithm estimates respiration and heart rates using remote photoplethysmography (rPPG). It processes video data to extract and analyze subtle changes in skin color caused by blood flow variations. The toolbox provides tools for detecting and tracking these physiological signals in real time. This non-invasive method offers an alternative to traditional monitoring techniques by leveraging standard video footage.	Python	Ubiquitous Computing Lab (University of Washington)
RESPIRATION_RATE_ESTIMATION [45]	The algorithm estimates respiration rates from video footage. It uses computer vision techniques to analyze the motion of the chest and abdomen, extracting features related to breathing. The algorithm processes these features to calculate the respiratory rate in real time. This approach offers a non-invasive method for monitoring respiration by leveraging visual data.	Python	Kapil Singh Rahtore (Indian Institute Of Tecnhology Madras)
Physio [46,47]	The repository is designed for processing and analyzing physiological signals, such as ECG and EEG. It provides tools for handling, preprocessing, and extracting features from these signals. The repository also includes visualization capabilities to aid in interpreting the data. It is intended to streamline the analysis of physiological data, potentially integrating with existing databases like PhysioNet.	Python	Samuel Garcia

## Data Availability

No new data were created or analyzed in this study. Data sharing is not applicable to this article.
